# Controlled Delivery of Vancomycin via Charged Hydrogels

**DOI:** 10.1371/journal.pone.0146401

**Published:** 2016-01-13

**Authors:** Carl T. Gustafson, Felix Boakye-Agyeman, Cassandra L. Brinkman, Joel M. Reid, Robin Patel, Zeljko Bajzer, Mahrokh Dadsetan, Michael J. Yaszemski

**Affiliations:** 1 Department of Molecular Pharmacology and Experimental Therapeutics, Mayo Graduate School, Mayo Clinic College of Medicine, Mayo Clinic, Rochester, Minnesota 55902, United States of America; 2 Pharmacometrics Center, Duke Clinical Research Institute (DCRI), Durham, North Carolina 27705, United States of America; 3 Division of Clinical Microbiology, Department of Laboratory Medicine and Pathology, Mayo Clinic Infectious Disease Research Laboratory, Mayo Clinic, Rochester, Minnesota 55902, United States of America; 4 Division of Biomathematics, Department of Biochemistry and Molecular Biology, Mayo Clinic College of Medicine, Mayo Clinic, Rochester, Minnesota 55902, United States of America; 5 Division of Biomathematics, Department of Physiology and Biomedical Engineering, Mayo Clinic College of Medicine, Mayo Clinic, Rochester, Minnesota 55902, United States of America; 6 Division of Orthopaedic Research, Department of Physiology and Biomedical Engineering, Mayo Clinic College of Medicine, Mayo Clinic, Rochester, Minnesota 55902, United States of America; Brandeis University, UNITED STATES

## Abstract

Surgical site infection (SSI) remains a significant risk for any clean orthopedic surgical procedure. Complications resulting from an SSI often require a second surgery and lengthen patient recovery time. The efficacy of antimicrobial agents delivered to combat SSI is diminished by systemic toxicity, bacterial resistance, and patient compliance to dosing schedules. We submit that development of localized, controlled release formulations for antimicrobial compounds would improve the effectiveness of prophylactic surgical wound antibiotic treatment while decreasing systemic side effects. Our research group developed and characterized oligo(poly(ethylene glycol)fumarate) / sodium methacrylate (OPF/SMA) charged copolymers as biocompatible hydrogel matrices. Here, we report the engineering of this copolymer for use as an antibiotic delivery vehicle in surgical applications. We demonstrate that these hydrogels can be efficiently loaded with vancomycin (over 500 μg drug per mg hydrogel) and this loading mechanism is both time- and charge-dependent. Vancomycin release kinetics are shown to be dependent on copolymer negative charge. In the first 6 hours, we achieved as low as 33.7% release. In the first 24 hours, under 80% of total loaded drug was released. Further, vancomycin release from this system can be extended past four days. Finally, we show that the antimicrobial activity of released vancomycin is equivalent to stock vancomycin in inhibiting the growth of colonies of a clinically derived strain of methicillin-resistant *Staphylococcus aureus*. In summary, our work demonstrates that OPF/SMA hydrogels are appropriate candidates to deliver local antibiotic therapy for prophylaxis of surgical site infection.

## Introduction

There have been recent reports that the risk of surgical site infection can be as high as 2.8% overall, with a rate up to 15% in some types of procedures.[1,2,3] This risk increases with the introduction of an implanted device due to biofilm formation on a new, easily colonizable surface. It seems reasonable to augment the standard intravenous prophylactic antibiotic regimen (which begins within 30 minutes of the incision) with local antibiotic treatment of the surgical wound in the immediate postoperative time period. There is *in vitro* evidence that bacterial colonization of implants occurs within hours of implant exposure to bacteria.[4,5] In an era when guarded use of antibiotics is prudent, it is essential to conservatively administer therapeutic doses of our diminishing stores of efficacious antimicrobial agents.[4,5]

The glycopeptide antimicrobial drug vancomycin was first isolated from *Amycolatopsis orientalis* and is broadly active against gram-positive bacteria.[6] Despite controversy over development of bacterial resistance and systemic toxicity, vancomycin use in clinical practice has gradually increased over recent years.[7] Cases of vancomycin resistant enterococci (VRE) have raised concerns over widespread vancomycin use.[8] In fact, some report that VREs may account for as many as 3% of hospital acquired infections.[9] In spite of these relatively sparse negative outcomes, vancomycin use has risen, while recent evidence suggests that bacterial resistance to vancomycin has not.[10] Treatment of fulminant infection of *C*. *difficile* relies heavily on the use and efficacy of both vancomycin and metronidazole.[11,12,13] Also, vancomycin is commonly indicated for treatment of cases of methicillin-resistant *Staphylococcus aureus*. Resistance to vancomycin among *S*. *aureus* microorganisms has been a promisingly rare occurrence, although this concern has gained traction in the past 20 years as a number of cases of *S*. *aureus* infection world wide have demonstrated a vancomycin minimum inhibitory concentration (MIC) greater than 100 μg/mL.[14] Serum concentrations of vancomycin at this level are not realistically achievable, thus, the vastly increased MIC characterizes strains of *S*. *aureus* that are not treatable by vancomycin. This notes a deviation from previously identified strains demonstrating “intermediate resistance” with MICs between 4–8 μg/mL. These cases exhibited limited response after treatment with vancomycin.[15]

Vancomycin use is most commonly limited by occurrences of thrombophlebitis and an immunologic adverse event known as “red man syndrome” due to systemic use. The efficacy of systemic vancomycin is also inhibited by unpredictable tissue exposure, as studies have shown that serum concentrations are poorly correlated to vancomycin levels within the tissue.[16,17] In surgical applications, delivery of concentrated doses of vancomycin to the wound site would bypass systemic, dose-limiting side effects. Indeed, many surgical teams have opted to use intra-operative powdered vancomycin as a wound dressing in an attempt to combat bacterial colonization of the wound.[18,19] This strategy has proved particularly successful in spine surgery, as a recent study has shown that odds of developing a deep wound infection following spine surgery decrease significantly with use of vancomycin powder.[20]

Pharmacokinetically, the efficacy of local vancomycin is inherently limited due to the high aqueous solubility and thus poor absorption and quick elimination of the drug. To control the local delivery of vancomycin, a bioresorbable, implantable delivery vehicle would be ideal. Recent research in biomaterials has yielded many such materials featuring a wide array of potential applications. Stimuli responsive hydrogels are water insoluble polymer networks containing ionic groups in the main chain or pendant regions. These materials are responsive to ionic strength, pH, temperature and electric field, among other stimuli. Responses to changes in environment can be predicted and controlled by the physical and chemical characteristics of the hydrogels.[21] Many of these responsive hydrogels have been previously reported for a variety of applications, such as molecule delivery (nucleic acids, proteins, or small molecules), cell encapsulation, enzyme/antibody immobilization, and protein separation or analytical techniques.[22,23,24,25,26,27,28,29,30] One proposed application is for the prophylaxis of infection post-surgery and prevention of bacterial fouling of implanted medical devices.[31] Responsive polymers as described above are excellent candidates to control dosing of an antibiotic for these applications–either as a coating of a medical device, an implantable gel, or as an injectable micro- or nano- particle formulation.[32,33,34]

One such polymeric system, oligo(poly(ethylene glycol)fumarate)/sodium methacrylate (OPF/SMA) co-polymer hydrogel films, has been used by our lab for drug delivery and tissue engineering purposes. It has been well characterized to be non-toxic and is able to be crosslinked into several known copolymer formulations.[35,36,37] The ability to produce a negatively charged hydrogel matrix led us to question if a positively charged molecule such as vancomycin would show an affinity for the hydrogel based on charge interactions. This affinity could be exploited as a tool to efficiently control vancomycin presence at the wound site. Antimicrobial delivery in this way would be highly beneficial to patients in order to avoid intravenous dosing while delivering a sustained, high concentration, localized dose to the surgical wound site.

## Materials and Methods

### OPF/SMA film fabrication

Oligo(poly(ethylene glycol)fumarate) macromer was synthesized as reported by Kinard, et al.[38] All reagents were obtained from Sigma-Aldrich, unless otherwise specified. The molecular weight of the polymer was measured by gel permeation chromatography on a Waters 717 Plus autosampler gel permeation chromatography system (Waters, Milford, MA) as previously reported by our group.[39] OPF/SMA hydrogel films were synthesized by methods previously described.[37,40] Briefly, 1 g of dried OPF was thoroughly mixed with 950 μl of double-distilled H_2_O and 750 μL of Irgacure® 2959 (BASF, Iselin, NJ) photoinitiator (10% mass / volume). The mixture was then incubated at 37°C for 10 minutes to completely dissolve the components. After incubation, 300 μL of 1-vinyl-2-pyrrolidinone (NVP) and the desired amount of sodium methacrylate powder were added to the mixture. These were mixed well to form a viscous solution, centrifuged to remove suspended bubbles and poured into molds before placing under UV light at room temperature. Crosslinking was achieved within 30 minutes. After UV crosslinking, the polymer film was cut into discs with a diameter of 3 mm. In order to remove any un-crosslinked material, the hydrogel films were dialyzed in Spectra/Por ® 6 Standard RC 50 kDa exclusion dialysis tubing for 72 hours. The films were then dried in a desiccating oven for 8 hours before use.

### Mammalian cell culture

Mouse bone marrow stromal fibroblasts (W20-17) and mouse pre-osteoblast (MC3T3-E1) cells were obtained from the American Type Culture Collection (ATCC). The cells were stored in liquid phase nitrogen prior to use. All cells were cultured in 75 cm^2^ flasks in Dulbecco’s Advanced Modified Eagles Media–F12 formulation. The cell cultures were passaged prior to confluency and to a total of less than six passages before final use.

### Cytotoxicity assay

W20-17 and MC3T3-E1 cells were plated at a cell density of 40,000 cells cm^-2^ in 12-well dishes 24 hours prior to addition of hydrogel discs. Vancomycin loaded hydrogel discs were sterilized in 100% ethanol prior to addition to tissue cultures. Hydrogel films (3 mm thick; 4.0–5.0 mg per sample) were placed in the wells of the dish such that the films were completely immersed in the culture media and in direct contact with the cells. Cell viability was measured by Cell Titer 96® MTS reagent assay (ProMega) after 72 hours of incubation at 37°C with the hydrogel films.

### Swelling Ratio

OPF / SMA hydrogels were synthesized as described. The hydrogels were dried, then incubated for 24 hours in either double distilled water, Dulbecco’s Phosphate Buffered Saline (DPBS), or a solution of vancomycin in double distilled water (400 μg/mL). The swollen hydrogels were dried briefly in room air, then under vacuum for 24 hours before weighing to determine the dried mass (Md). The samples were then swollen again for 24 hours in their respective solutions, and weighed again to determine swollen mass (Ms). The swelling ratio (SR) was calculated as the dried mass (Md) subtracted from the swollen mass (Ms) and divided by the dried mass, such that SR = (Ms-Md) / Md.

### Thermal Characterization

Thermogravimetric analysis (TGA) was performed using a TA Instruments Q Series™ Discovery Thermogravimetric Analyzer at a heating ramp rate of 20°C min^-1^ from 50°C to 1000°C under an inert atmosphere. Differential scanning calorimetry (DSC) of the copolymer hydrogels was performed under an inert atmosphere in a TA Instruments Q Series™ Discovery Differential Scanning Calorimeter. Samples were equilibrated to 100°C, cooled to -80°C at a rate of 5°C min^-1^, then heated to 150°C at a rate of 5°C min^-1^.

### High Performance Liquid Chromatography (HPLC)

We developed a facile HPLC method for detection and quantitation of vancomycin. This method involved the use of a gradient elution on a reversed-phase Aquasil™ C18 column (150 x 4.6 mm i.d., 2.1 μm particle size, Thermo Fisher Scientific, Hudson, New Hampshire), and an ultraviolet detection at a single wavelength (282 nm). The mobile phase consisted of dibasic potassium phosphate buffer (5 mM, pH = 2.8) and acetonitrile. The HPLC system consisted of a Shimadzu liquid chromatograph (Wood Dale, IL, USA) with one LC-20 AD quaternary pump (flow rate 0.5 mL/min), a DGU-14 degasser, and a SIL-10ADvp controller. The retention time of vancomycin was 4.9 minutes. Concentrations of vancomycin in sample solutions were determined by comparison to a linear graph of vancomycin concentrations based upon the areas under the peaks of prepared standard concentrations.

### OPF/SMA hydrogel drug loading

Dried hydrogel films were incubated in 1 mL of a 400 μg/mL (unless otherwise specified) vancomycin hydrochloride solution for 24 hours (unless otherwise specified) at ambient temperature. The films were then removed, placed on a dish, and desiccated. The final concentration of the vancomycin in each sample tube was measured by high pressure liquid chromatography after incubation. The total amount of drug loaded onto each hydrogel film was measured by subtracting the final concentration of drug from the initial concentration of drug and multiplying by the total volume of the solution ([VCM]_i_−[VCM]_f_)*V_t_ = mass_loaded_. Loading efficiency was calculated as the unit mass of vancomycin achieved per unit mass of hydrogel (i.e. μg VCM/mg hydrogel).

### OPF/SMA hydrogel drug release

Loaded OPF/SMA hydrogel films were desiccated prior to initiation of drug release. Dry hydrogel films were placed in 1 mL of Dulbecco’s Phosphate-Buffered Saline (DPBS) in a low retention 2 mL centrifuge tube and incubated at 37°C for the entire release period. Solutions of DPBS were changed at each time point (6, 12, 24, 36, 48, 72, and 96 hours), and drug concentrations were measured immediately after collection to avoid error due to drug degradation during storage. In order to control for degradation of drug in solution over the release period, stock concentrations of vancomycin were stored at 37°C during the release period and used for standards in HPLC measurements. Individual release concentrations were measured using the established HPLC assay described above in Materials and Methods. Data points represent cumulative release of drug over the accrued time period. Error bars shown are standard deviations of 3 measurements for the individual release interval that was measured (non-cumulative). Amount of drug released was calculated by measuring the concentration of drug within the sample and multiplying by the volume of the solution that the sample was eluting into (1.0 mL) ([mass / volume] * volume). All measurements were repeated twice, n = 3 within each experimental group.

### Mathematical modeling

We analyzed the vancomycin release data using a phenomenological mathematical model based on a stretched exponential function in a manner that we previously found promising.[40] The cumulative release F(t) is given by F(t) = C[1 –exp(-(t/τ)^b^], according to that model, where C is the maximally achieved cumulative release (100%) and τ is the time when (1-e^-1^)C = 0.632C is achieved. Parameter b defines the stretching of the exponential function. T_1/2_, or the time when half of the total released drug had been released, was calculated by the equation T_1/2_ = τ(ln 2)^1/b^. T_1/2_ and τ indicate time to 50% release and 63% release, respectively, while r is defined as the slope of the line tangent to the curve at time T_1/2_ and represents the instantaneous release rate at T_1/2_. To further characterize the cumulative release we also calculated the instantaneous release rate at T_1/2_ (the slope of the line tangent to the curve at time T_1/2_), given as r = Cb(ln2)/(2T_1/2_).

### Microorganism

Methicillin-resistant *Staphylococcus aureus* (IDRL-9658), a vancomycin susceptible clinical isolate recovered from a prosthetic knee infection, was studied. The MIC of vancomycin for this bacterium was 1 μg/mL vancomycin.

### Bacterial killing assay

*S*. *aureus* was prepared to 0.5 McFarland in tryptic soy broth (TSB) and diluted 1:200 in TSB. 100 μl of bacterial culture was placed into each well in a 96 well plate. 100 μl of each experimental sample was added to the bacterial culture for final concentrations of 2.5, 5 and 10 μg/mL hydrogel-eluted vancomycin. Control wells were performed using bacterial culture with commercially available (“stock”) vancomycin at the same concentrations. In addition, wells containing no bacteria and wells containing bacteria treated with saline only were prepared in parallel to serve as growth controls. Plates were incubated under static conditions at 37°C for 24 hours in room air. Following growth for 24 hours, quantitative cultures were performed using serial dilutions and grown on BBL™ Trypticase™ Soy Agar with 5% Sheep Blood (Becton Dickinson, Franklin Lakes, New Jersey). Plates were incubated for 24 hours at 37°C in CO_2_. Following incubation the log_10_ colony forming unit (cfu)/ml was calculated. Bacterial killing of eluted solutions was compared to the bacterial killing of stock solutions. Results of each experiment are from a representative set of n = 3 specimen groups. Each experiment was then performed in duplicate.

### Zone of clearing assay

*S*. *aureus* was prepared to 4 McFarland in 5 ml of saline. 1 ml of this solution was added to 100 ml of MHA agar and allowed to solidify. Charged or uncharged hydrogels with or without vancomycin were placed on to the plates and incubated at 37°C in room air for 24 hours. Zones of clearing (mm) for each hydrogel sample were measured using a calipers. Each group was done in triplicate.

### Statistical Analysis

Two-tailed unpaired t-test, or one-way analysis of variance (ANOVA) was applied, when applicable, for the statistical analysis of data from each group in this study. Groups that were calculated with p-value lower than 0.05 (p<0.05) were considered to have statistical difference.

## Results

### OPF/SMA hydrogels are biocompatible

**Fig 1:**
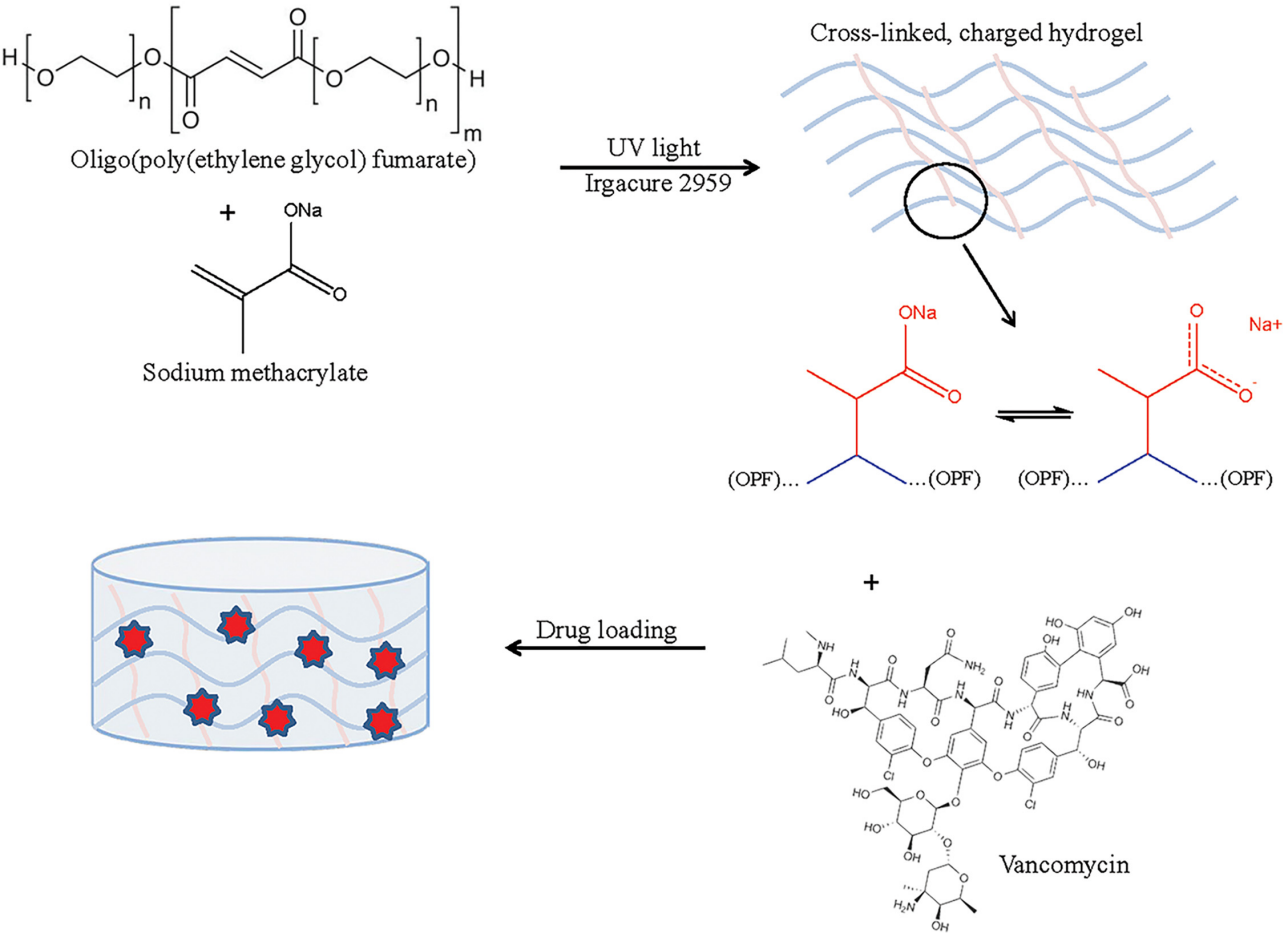
Schematic depicting synthesis of OPF/SMA hydrogels. OPF/SMA hydrogels synthesized as described. Idealized OPF chain represented in blue, SMA is depicted in red, crosslinked between OPF chains. Vancomycin shown as red stars within the hydrogel.

**Fig 2:**
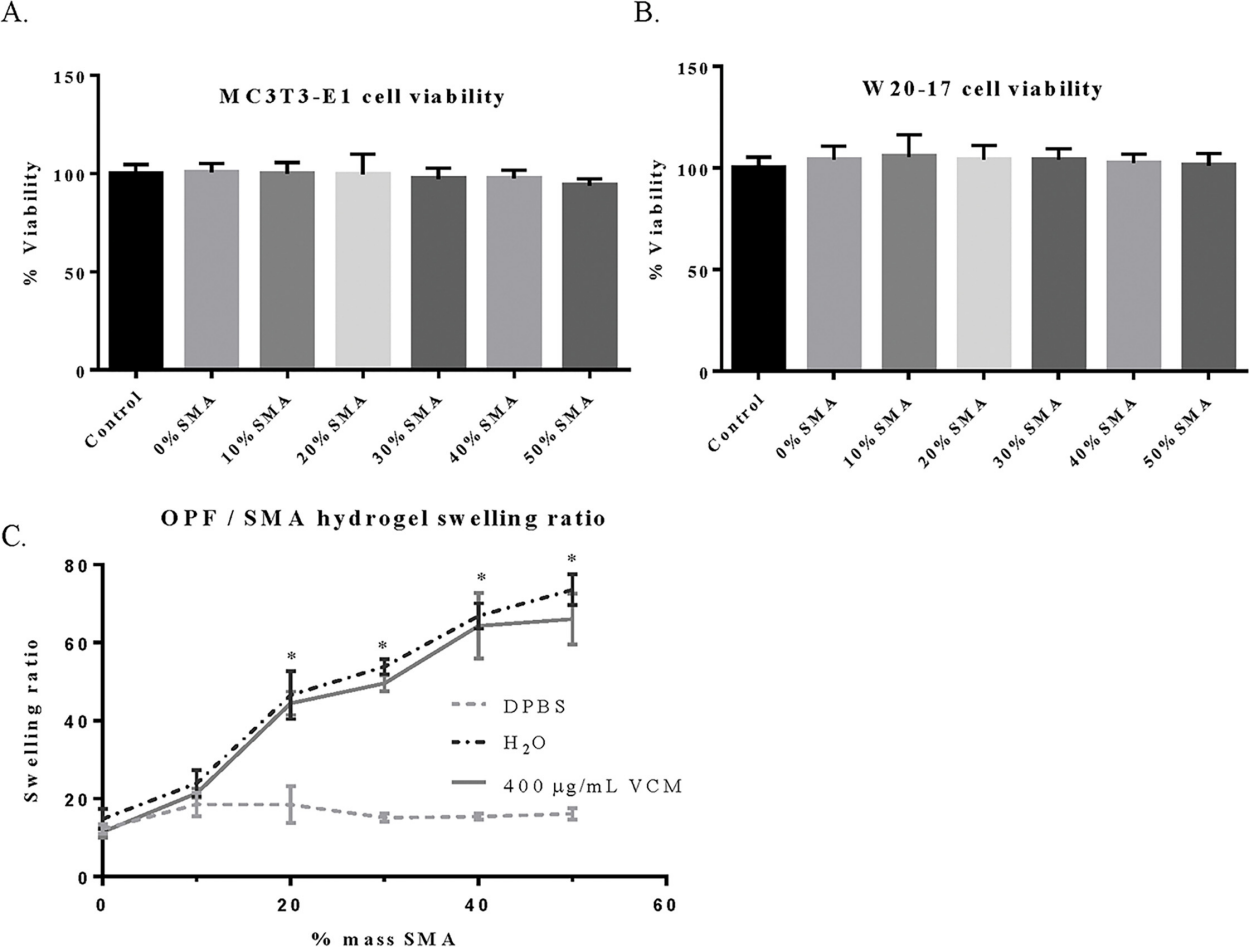
Biocompatibility and swelling ratio of OPF/SMA hydrogel discs. Cell viability of MC3T3-E1 (A) and W20-17 (B) cells incubated with OPF/SMA copolymer hydrogel films. Cell viability determined after 72 hour incubation in the presence of OPF/SMA hydrogel discs. Viability assessed with CellTiter Glo 96 MTS viability assay (ProMega). Hydrogels were directly placed into the dishes and were in contact with cell cultures during the incubation time. Hydrogel discs (as described) were swollen in either, double distilled water, DPBS, or a solution of vancomycin hydrochloride (400 μg/mL) in double distilled water. The hydrogels were dried, weighed, then swollen again and weighed again. Swelling ratio (SR) calculated as described in Materials and Methods. Error bars represent +/- one standard deviation, N = 3 in all groups. (*) indicates a statistically significant difference (p<0.05) between DPBS and double distilled water groups.

A series of OPF/SMA hydrogels discs were synthesized as described (Fig 1), with increasing percent by mass SMA. OPF/SMA hydrogel discs are non-toxic in cell culture (Fig 2A and 2B). Biocompatibility of the OPF/SMA hydrogel films was demonstrated by incubation of hydrogel samples for 72 hours in direct contact with the mouse pre-osteoblast or fibroblast cell lines MC3T3-E1 or W20-17, respectively. Cell viability was measured by MTS assay. With all OPF/SMA copolymer formulations, we saw no toxicity at any time in the cell lines tested. These cell lines were chosen for *in vitro* experimentation in order to test the plausibility of the use of the OPF/SMA hydrogels in a mouse model of surgical site infection following orthopaedic surgery.

### Characterization of biophysical and thermal properties

OPF/SMA hydrogel films were synthesized by UV crosslinking. Interestingly, a distinct trend in swelling ratio correlated with increased SMA content in the copolymer was observed. In DPBS, the swelling ratio of the various hydrogel formulations remained relatively constant throughout. In double distilled water or vancomycin solution in double distilled water, swelling ratio increased significantly with increased SMA content (Fig 2C). No statistically significant difference was found between swelling in water and swelling in vancomycin solution. The increased SMA content hydrogels contain a higher density of ionic interactions which dissociate freely within a non-ionic solution (e.g. water or low-concentration vancomycin solution). This allows for greater swelling due to the osmotic pressure of the charged, crosslinked SMA. In an ionic solution (DPBS), the various hydrogel formulations act similarly, as the high concentration of free ions in solution cause the dissociation of SMA to be less favorable. Thermogravimetric analysis (TGA) and differential scanning calorimetry (DSC) showed that changes in OPF/SMA ratio did not dramatically change the degradation temperature of the copolymer. Our DSC analysis found that the enthalpy of crystallization and the enthalpy of fusion decreased dramatically in higher SMA content hydrogels. Although TGA thermal properties remained relatively constant between groups, hydrogels with increased SMA content demonstrated additional and increasingly significant mass losses between 60–140°C and 800–890°C (S1 Table; S1A and S1B Fig). Strikingly, enthalpy of crystallization, enthalpy of fusion, and percent crystallinity decreased dramatically in higher percent SMA samples. We attribute this finding to changes in hydrogel structure with increased SMA incorporation (Table 1).

**Table 1:** Thermal properties of OPF/SMA copolymer hydrogels.

	T_c_ (°C)	ΔH_c_ (J/g)	T_m_ (°C)	ΔH_m_ (J/g)	%c	T_d_
**Uncrosslinked OPF**	41.90	116.80	57.80	130.90	63.50	335.00
**OPF**	32.75	169.28	52.46	180.05	87.49	422.94
**OPF/SMA10**	31.43	162.46	50.53	162.84	79.12	429.88
**OPF/SMA20**	32.32	82.78	50.71	84.24	40.93	428.23
**OPF/SMA30**	31.60	62.47	49.82	59.09	28.71	427.98
**OPF/SMA40**	28.83	56.09	47.49	55.38	26.91	431.41
**OPF/SMA50**	28.57	45.22	47.46	43.71	21.24	429.29

Differential scanning calorimetry used to determine temperature of crystallization (T_c_), enthalpy of crystallization (ΔH_c_), melting temperature (T_m_) and enthalpy of fusion (ΔH_m_). Percent crystallinity (%c) calculated using ΔH_m_ of starting material PEG 10,000 = 205.81 (J/g).[41] Thermogravimetric analysis used to determine degradation temperature (T_d_). Parameters for analytical methods outlined in Materials and Methods section.

### Kinetics of vancomycin loading onto OPF/SMA hydrogel films

**Fig 3:**
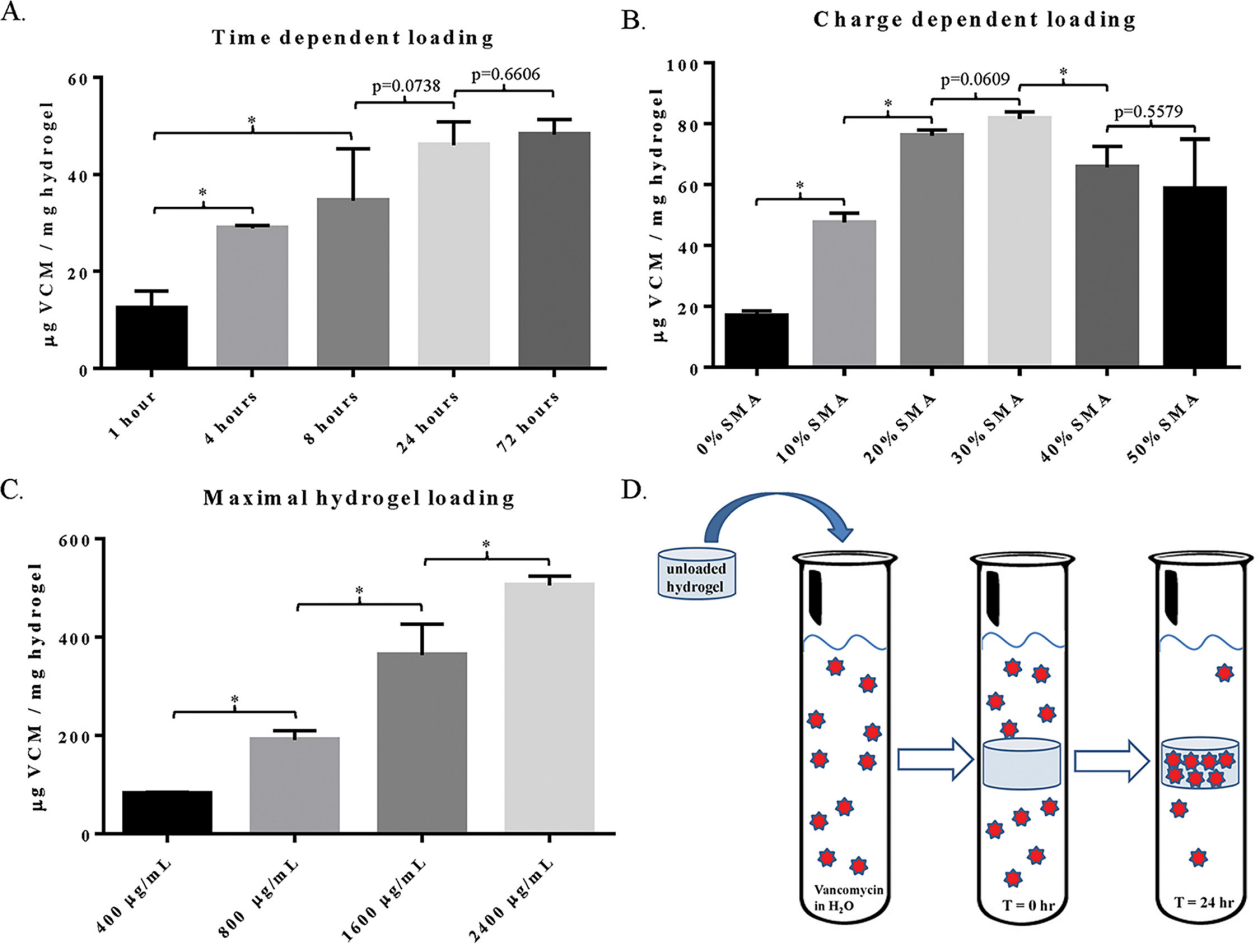
Analysis of vancomycin loading in OPF/SMA hydrogels. (A) OPF/SMA 40% hydrogels were incubated with vancomycin at 400 μg/mL for 1 hour, 4 hours, 8 hours, 24 hours, or 72 hours. The concentrations of vancomycin in solution were then measured with HPLC coupled to UV-Vis detection at 280 nm and drug loading was determined by subtracting the initial concentration of drug from the final concentration after completion of vancomycin loading. (B) Hydrogels were incubated for 24 hours, samples were collected, and the concentration of vancomycin in solution was determined as described. Amount of drug loaded was calculated and loading efficiency was determined by dividing the mass of loaded drug by the dried hydrogel mass, such that loading efficiency is represented as μg vancomycin per mg hydrogel. (C) Maximal drug loading was determined by incubating OPF/SMA 30% hydrogel samples in increasingly concentrated solutions of vancomycin in double distilled water for 24 hours. Loading efficiency (Eff_l_) was determined by measurement of the final drug concentration in the distilled water (C_f_) after completion of the loading cycle, followed by comparison to concentration of initial solution (C_i_) via the equation (Eff_l_) = {[(C_i_)—(C_f_)] / (C_i_)}*100%. (D) Diagram representing drug loading experiment. In all experiments, concentrations of vancomycin remaining were determined from a standard curve of vancomycin solution that was incubated under identical conditions to the sample of interest. Error bars represent +/- one standard deviation, N = 3 in all groups. Statistical representation: (*) indicates p<0.05.

Drug loading in 400 μg/mL vancomycin at room temperature and pressure increased with time for up to 72 hours (approx. 22°C, 1 atm). However, maximal loading was observed at 24 hours, and extending the loading period beyond this time did not significantly increase loading efficiency (Fig 3A). We found that loading efficiency increased significantly with increased SMA content in the hydrogel formulations, indicating that free charges in the pendant groups of the hydrogel matrix are essential for binding vancomycin and pulling it out of solution (Fig 3B). This trend continued up to the 30% SMA copolymer, with a sudden drop in loading efficiency in the 40% and 50% SMA groups. We hypothesize that this phenomenon is due to saturation of charge in the copolymer matrix, where excess free charges self-inhibit binding of the drug molecules to each other. Vancomycin loading is then mediated by this ionic interaction between charges on the hydrogel and charged groups available on the drug. Higher concentrations of SMA crosslinked into the OPF hydrogel are possible (e.g. 40% and 50% SMA) but the loading efficiency begins to decline precipitously at those levels. We attempted to load the OPF/SMA hydrogel films with vancomycin until they were saturated (maximum loading) but were unable to do so. We saw that at high concentrations of drug (up to 2.4 mg/mL vancomycin), the hydrogel film was able to bind vancomycin at the same loading efficiency (% of available drug that could be loaded) as at low concentrations (400 μg/mL) (Fig 3C). We were able to achieve loading efficiencies of vancomycin over 500 μg vancomycin per mg of hydrogel, where vancomycin accounted for greater than 1/3 of the total mass of the loaded hydrogel (Fig 3C). In a complementary approach, we utilized TGA to detect the percent mass accounted for by vancomycin in the hydrogel (S2D Fig). This method proved the incorporation of vancomycin into the hydrogels. We found that these data correlated strongly with the data obtained by measuring loading efficiency with our HPLC method, further strengthening our results from the loading experiment.

### Vancomycin release controlled by sodium methacrylate interaction

**Fig 4:**
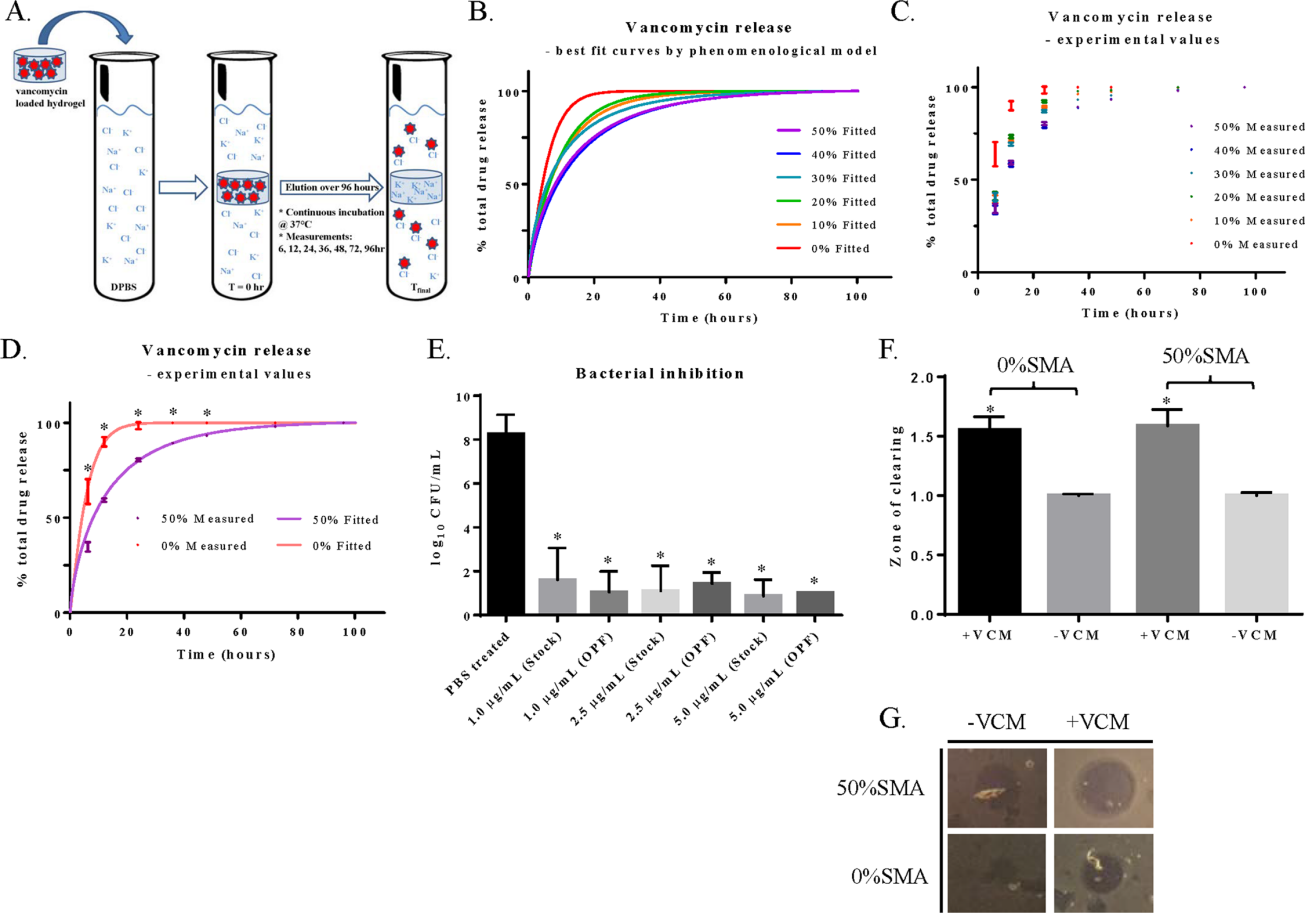
Vancomycin release from OPF/SMA hydrogels. (A) Diagram representing release of vancomycin from charged hydrogels. OPF/SMA hydrogels loaded with vancomycin were placed in DPBS solutions and incubated at 37°C for 96 hours. Solutions were collected and fresh DPBS was replaced at the indicated time points. The concentration of eluted vancomycin was measured by HPLC. Cumulative release is represented as a percentage of the total amount of measured drug released. (B) Best fit of data as calculated by phenomenological mathematical model described in text. (C) Experimentally measured data points obtained, plotted for comparison to mathematical model. (D) Comparison of fitted model to obtained data points for 0%SMA and 50%SMA hydrogels. (E) Efficacy of released drug was determined by treating a strain of MRSA (MIC_50_ for vancomycin = 1.0 μg/mL) with solutions of released drug from the 12 hour sample group. Stock concentrations of vancomycin hydrochloride were compared to released drug at equivalent concentrations of 5.0, 2.5, or 1.0 μg/mL. Efficacy tested in a quantitative colony forming assay and compared to PBS treated control. (F) Zone of clearing assay comparing vancomycin loaded and unloaded hydrogels (0%SMA or 50%SMA). Values normalized to vancomycin unloaded hydrogels (zone of clearing = 1). (G) Representative images of hydrogels ability to clear bacteria from a dish. Error bars represent +/- one standard deviation, N = 3 in all groups. Statistical representation: (*) indicates p<0.05.

Vancomycin release from OPF/SMA hydrogels is mediated by ion exchange. Because vancomycin attraction to the hydrogel matrix appears to be mediated by charge interactions, we hypothesized that in an ionic solution such as saline or DPBS, the buffering of these charges by high ion concentrations within the solution would cause vancomycin to release from the hydrogel film. We desiccated hydrogel films that had been loaded with vancomycin, and placed them in solutions of DPBS. The hydrogels were incubated at 37°C for 96 hours, with measurements of drug release taken at 6, 12, 24, 36, 48, 72, and 96 hours (Fig 4A). We found that vancomycin was released from the hydrogel into solution when the hydrogels were placed in DPBS. The initial (burst) release of vancomycin from the hydrogel was dampened by increasing the charge density within the hydrogel matrix (increased SMA). We observed that hydrogel films without SMA had a much higher burst release–as high as 90% in the first 12 hours compared to 58% in the 10%, 20%, and 30% SMA groups (Fig 4B and 4C, S3 Fig). The experimental groups that had higher concentrations of SMA (40% and 50%) released the vancomycin more slowly, and demonstrated extended release up to and past 4 days (5.7 and 5.1 μg/mL at t = 96 hrs in the 40% and 50% SMA groups, respectively), while having a lower initial burst release (Fig 4D and 4E). This delayed release positively correlates with increased charge–charge interactions between the hydrogel film and the vancomycin. We observed that release of physiologically relevant concentrations of the loaded drug is possible as far as 4 days. The target serum concentration of vancomycin in patients is 15 μg/mL.[42] This number does not accurately reflect availability of vancomycin within deeper tissue, or at an ischemic wound site as interstitial concentrations are lower and depend on efficiency of tissue perfusion within the biological context. Thus, for an implanted material such as ours, an efficacious dose is not clearly predicted by serum concentration standards (which are established for systemic dosing, compared to local). We propose, then, that the minimum inhibitory concentration (MIC) of a given bacterial strain represents a more robust prediction of antibiotic efficacy.

### Mathematical modeling of vancomycin release

A phenomenological mathematical model based on a stretched exponential function provided a very good fit for all data (Fig 4C and S3A and S3B Fig). The estimated values of T_1/2,_ τ and r, together with estimates of b, are shown in Table 2. The uncertainties of the estimated parameters in Table 2 are calculated by a Monte Carlo method based on the standard deviations in the data set.[43] It is of interest to observe that τ and T_1/2_ have the smallest value for 0% SMA, which is to be expected, then practically the same value for 10% SMA, 20% SMA and 30% SMA, which is less expected (Table 2). For 40% SMA, τ and T are maximal, while for 50% they are slightly decreased. That would indicate that 40% SMA is perhaps the optimal condition for minimizing the rate of drug release. The value of r (the instantaneous release rate) is similarly the lowest for 40% SMA, and only slightly increased for 50% SMA. In regard to parameter b, one can see the significant drop for 30, 40, and 50% SMA in comparison to 0%, 10% and 20% SMA, which also indicates decreased release kinetics. The smaller the value for parameter b, the slower is the convergence to C.

**Table 2:** Summary of parameters derived by mathematical modeling of vancomycin release.

	τ	T_1/2_	r	b
**0%SMA/OPF**	5.84 +/- .73	4.29 +/- .76	9.70 +/- .81	1.120 +/- 0.25
**10%SMA/OPF**	9.53 +/- .20	6.34 +/- .18	4.92 +/- .057	0.899 +/- 0.016
**20%SMA/OPF**	9.23 +/- .20	6.36 +/- .18	5.35 +/- .064	0.982 +/- 0.017
**30%SMA/OPF**	9.51 +/- .25	5.76 +/- .21 ^ᵵ^	4.42 +- .069 ^ᵵ^	0.730 +/- 0.017 ^ᵵ^
**40%SMA/OPF**	14.1 +/- .22 ᴻ	8.96 +/- .22 ᴻ	3.15 +/- .019 ᴻ	0.806 +/- 0.017 ᴻ
**50%SMA/OPF**	13.3 +/- .24 *	8.23 +/- .23 *	3.26 +/- .025 *	0.767 +/- 0.018 *

*Tau (τ)* represents time at which ~63% drug has been released. *T*_*1/2*_ represents time at which 50% drug has been released. *r* represents the slope of a tangent line at time = T_1/2_. *b* represents the stretching of the exponential curve. Uncertainties were determined by the Monte Carlo method, N = 250. Statistical representation

(^ᵵ^) indicates p<0.05, for 30%SMA/OPF compared to 10%SMA/OPF.

(ᴻ) indicates p<0.05, for 40%SMA/OPF compared to 20%SMA/OPF.

(*) indicates p<0.05, for 50%SMA/OPF compared to 0%SMA/OPF.

### Vancomycin remains biologically active once released

We considered the possibility that the released vancomycin could be biologically inactivated by chemical modification after the loading/incubation/release cycle within the hydrogel film. In order to answer this question, we obtained a strain of methicillin-resistant *Staphylococcus aureus* (MRSA) that originated from a prosthetic joint infection (courtesy of Dr. Robin Patel, Infectious Disease Research Laboratory, Mayo Clinic, Rochester, MN). We treated the MRSA cultures with solutions of vancomycin that had been released from the hydrogels (12 hour release solutions), and found that after the vancomycin eluted from the hydrogel films it inhibited the growth of bacterial colonies on agar plates (Fig 4D). The inhibition of bacterial growth by the hydrogel-eluted vancomycin was equivalent to the inhibition of bacterial growth by stock vancomycin solutions that had not been loaded and released from the OPF hydrogels. We also assessed the ability of the drug-loaded hydrogels to inhibit the growth of bacteria in a zone of clearing assay. We compared the clearing ability of 0%SMA (vancomycin loaded and unloaded) and 50%SMA (loaded and unloaded) hydrogels which had been allowed to release drug for 24 hrs (Fig 4F and 4G). We found that after 24 hours of drug release, the vancomycin loaded hydrogels (both 0% and 50% SMA groups) were able to efficiently clear bacteria from the plate. The clearing ability of 0% and 50% SMA groups was equivalent at this time point, which was to be expected due to the high concentrations of drug which remained within both groups of hydrogel at the 24 hour time point.

## Discussion

Despite improved surgical procedures and increased awareness of factors that may increase the risk of surgical site infection, the rate of surgical site infection remains near 1–3% during clean surgical procedures. Bone grafting, spinal reconstruction with instrumentation, tissue reconstruction procedures, and joint arthroplasty are at particular risk, due to the presence of new biologic or prosthetic surfaces susceptible to bacterial colonization. More than half of the prosthetic joint infections in the Unites States are caused by staphylococci.[44] Hip and knee related prosthetic joint infections are projected to incur a yearly monetary burden of 1.62 billion USD by the year 2020, having risen from 320 million USD to 566 million USD between 2001 to 2009.[45] Sessile bacteria are inherently more resistant to antimicrobial therapies due to the mechanical properties of the biofilm they secrete, as compared to planktonic bacteria of the same species.[46,47] Our ability to prevent biofilm development will determine our ability to improve patient outcomes, retain effective antibiotics and lower the economic burden due to infection. As bacterial resistance to commonly used antimicrobials increases, an ever shortening list of potent antibacterial agents will be available. Judicious use of these agents is imperative in order to continuously provide patients with cost-efficient and functional antimicrobial therapies.

This research is specifically targeted at addressing the need for localized, high dose antibiotic concentrations at the surgical site immediately following surgery–an effort that will complement existing measures in place to prevent infection. Stimuli-responsive polymer systems have been an area of intense research resulting in the development of methods for cell encapsulation and delivery techniques, protein identification, immune targeting and drug release.[25,28,30] Indeed, recent studies have reported both *in vitro* and *in vivo* use of hydrogels for the extended delivery of antibiotics.[48,49,50] The present work represents an additional step forward in this field, as we demonstrate the malleable nature of OPF/SMA hydrogels’ loading and release properties. Further, the simplicity of the charge based interactions enables facile loading of drug and limits preparation time, which will prove to be a significant advantage in future clinical applications. The OPF/SMA hydrogels that we have developed are currently not suited for extended delivery of drug. In contrast, many studies have demonstrated successful local release reaching to further time points. We believe that the application of our hydrogel system is relevant for prevention of rapid onset surgical site infection, which has been reported to begin within hours.[4] Our study demonstrates that oligo(poly(ethylene glycol)fumarate)/sodium methacrylate (OPF/SMA) co-polymer hydrogels may be a useful tool in the surgical setting for antimicrobial agent delivery. Our data show that these hydrogels are non-toxic and have predictable thermo-physical properties. The charge-dependent characteristics of this drug release mechanism accent its highly tunable nature.

Although our study did not explore specific optimization of OPF/SMA hydrogel release kinetics, we demonstrate that the drug release rate is predictably correlated to hydrogel structure. The mathematical model we have developed will be of great utility for further development and optimization of this antibiotic delivery vehicle. While the target serum concentration of vancomycin in patients is 15–20 μg/mL, this range does not accurately reflect the availability of vancomycin within deeper tissue, or at an ischemic wound site.[16,42,51] Thus, antibiotic efficacy may be predicted more accurately by the MIC of a given bacterial strain. For any local delivery vehicle such as ours, extensive *in vivo* characterization would be necessary in order to identify optimal loading and release properties. It is very likely that efficacious intra-tissue concentrations of drug may be much lower than serum concentrations, and release kinetics will almost certainly be variable depending on implant location. The strength of our hydrogel system is its ability to be tailored to fit application-specific needs. Our study was limited to investigation of the properties of OPF/SMA hydrogel films alone. As OPF/SMA hydrogels can be synthesized into microsphere/nanosphere formulations, an injectable, extended release delivery method could readily be designed and fabricated. Also, these particulate formulations could be incorporated into a second polymer if the need for improved structural properties arises.

Here, we show that OPF/SMA matrices are capable of extended release of vancomycin *in vitro*, while retaining the biological efficacy of the released drug. We conclude that this delivery vehicle is an excellent candidate for use in local elution of vancomycin for surgical site infection prophylaxis. Future research will involve the investigation of the *in vivo* pharmacokinetics of vancomycin delivery from OPF/SMA hydrogels, and the implementation of this drug delivery vehicle for decreasing the surgical site and prosthetic joint infection rates in rodent models.

## Supporting Information

S1 Fig DSC and TGA overlays from OPF/SMA hydrogels. (A) DSC overlay: crystallization and melting temperatures labelled. (B) TGA overlay: Thermal characterization of OPF / SMA hydrogels. Significant mass losses labelled as T1, T2, and T3.
(TIF)

S2 Fig Representative HPLC graphs and correlation of TGA results to HPLC data. A. Blank sample used in the standard curve for the 24 hour time point. B. Vancomycin standard at 10 μg/mL used in the standard curve for the 24 hour time point. Retention time = 4.925 minutes, concentration of the sample = 9.685 μg/mL (back-calculated based off the standard curve). C. Sample 50B from a drug loading experiment, drug concentration determined from a set of drug standards handled and ran in parallel to the sample solutions. D. Thermogravimetric analysis of vancomycin loaded OPF/30%SMA hydrogel films. The percentage of the initial mass that remained at 1000°C was observed to increase linearly with increased vancomycin loading at higher concentrations of drug. With unloaded hydrogels, an insignificant mass remained at 1000°C. The percentage of the initial mass that remained at 1000°C correlated significantly with drug loading efficiency (R^2^ = 0.9889). Loading concentrations of 400, 800, 1600 and 2400 μg/mL were tested, N = 2 for all groups.
(TIF)

S3 Fig Fitted curves of vancomycin release data. (A) Comparison of fits for 10% SMA/OPF and 30% SMA/OPF hydrogel vancomycin release. The light colored solid lines are best fit curves based on mathematical model described in text. The experimentally obtained data with error bars (some error bars not visible at this scale) are shown by darker points. (B) Comparison of fits for 20% SMA/OPF and 40% SMA/OPF hydrogel vancomycin release.
(TIF)

S1 Table Thermogravimetric characterization of OPF/SMA hydrogel films. Analysis of TGA scans and compilation of average degradation temperatures. Values are a mean calculated from replicate experiments (N = 2). Undetectable value denoted by (N/A).
(TIF)
