# Supplementary figures and images for: Controlled Delivery of Vancomycin via Charged Hydrogels
Source: PLoS One. 2016 Jan 13;11(1):e0146401. doi: 10.1371/journal.pone.0146401 (PMC4711919; doi:10.1371/journal.pone.0146401)

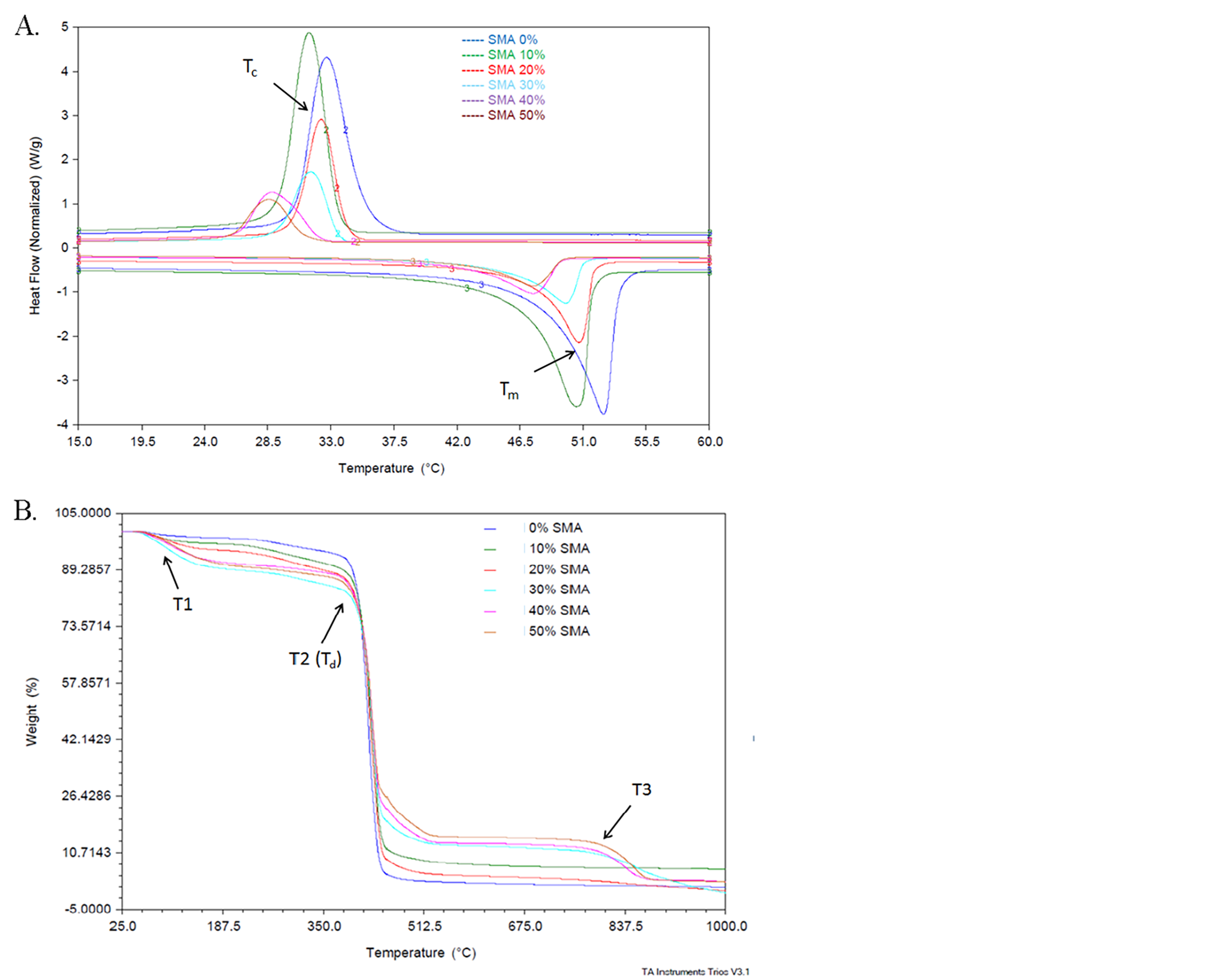

Supplement: S1 Fig — (A) DSC overlay: crystallization and melting temperatures labelled. (B) TGA overlay: Thermal characterization of OPF / SMA hydrogels. Significant mass losses labelled as T1, T2, and T3. (TIF) [file pone.0146401.s001.tif]

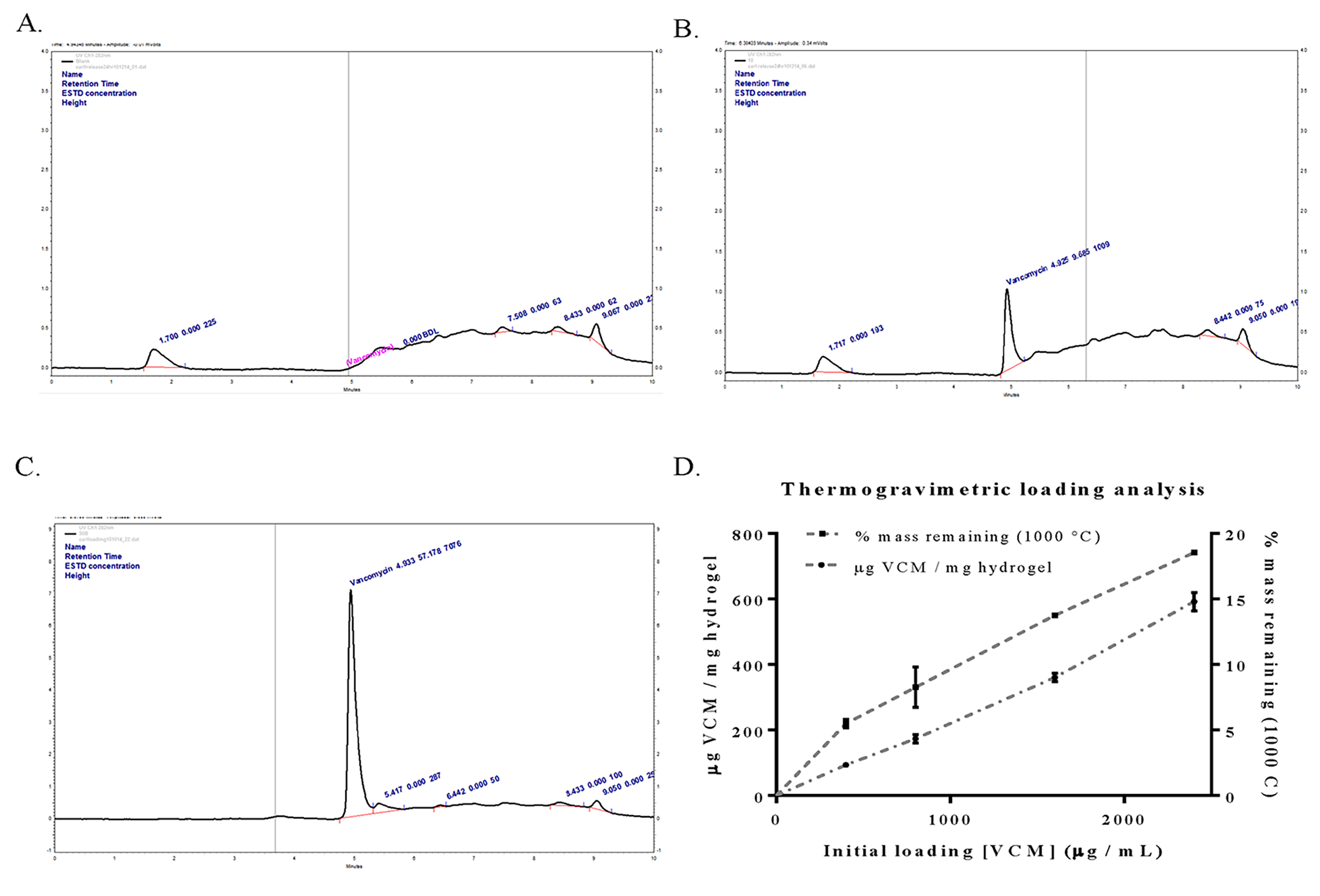

Supplement: S2 Fig — A. Blank sample used in the standard curve for the 24 hour time point. B. Vancomycin standard at 10 μg/mL used in the standard curve for the 24 hour time point. Retention time = 4.925 minutes, concentration of the sample = 9.685 μg/mL (back-calculated based off the standard curve). C. Sample 50B from a drug loading experiment, drug concentration determined from a set of drug standards handled and ran in parallel to the sample solutions. D. Thermogravimetric analysis of vancomycin loaded OPF/30%SMA hydrogel films. The percentage of the initial mass that remained at 1000°C was observed to increase linearly with increased vancomycin loading at higher concentrations of drug. With unloaded hydrogels, an insignificant mass remained at 1000°C. The percentage of the initial mass that remained at 1000°C correlated significantly with drug loading efficiency (R2 = 0.9889). Loading concentrations of 400, 800, 1600 and 2400 μg/mL were tested, N = 2 for all groups. (TIF) [file pone.0146401.s002.tif]

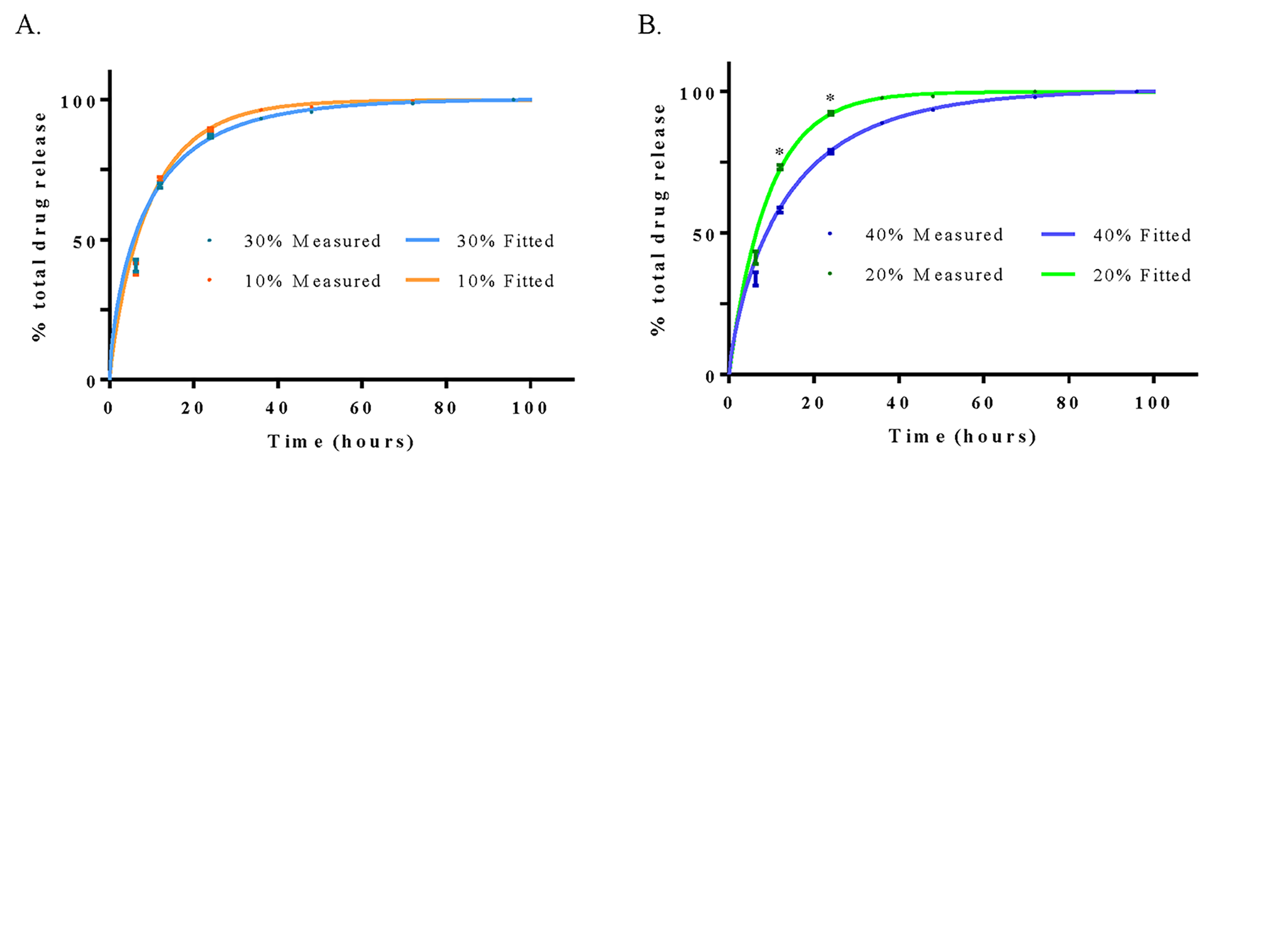

Supplement: S3 Fig — (A) Comparison of fits for 10% SMA/OPF and 30% SMA/OPF hydrogel vancomycin release. The light colored solid lines are best fit curves based on mathematical model described in text. The experimentally obtained data with error bars (some error bars not visible at this scale) are shown by darker points. (B) Comparison of fits for 20% SMA/OPF and 40% SMA/OPF hydrogel vancomycin release. (TIF) [file pone.0146401.s003.tif]

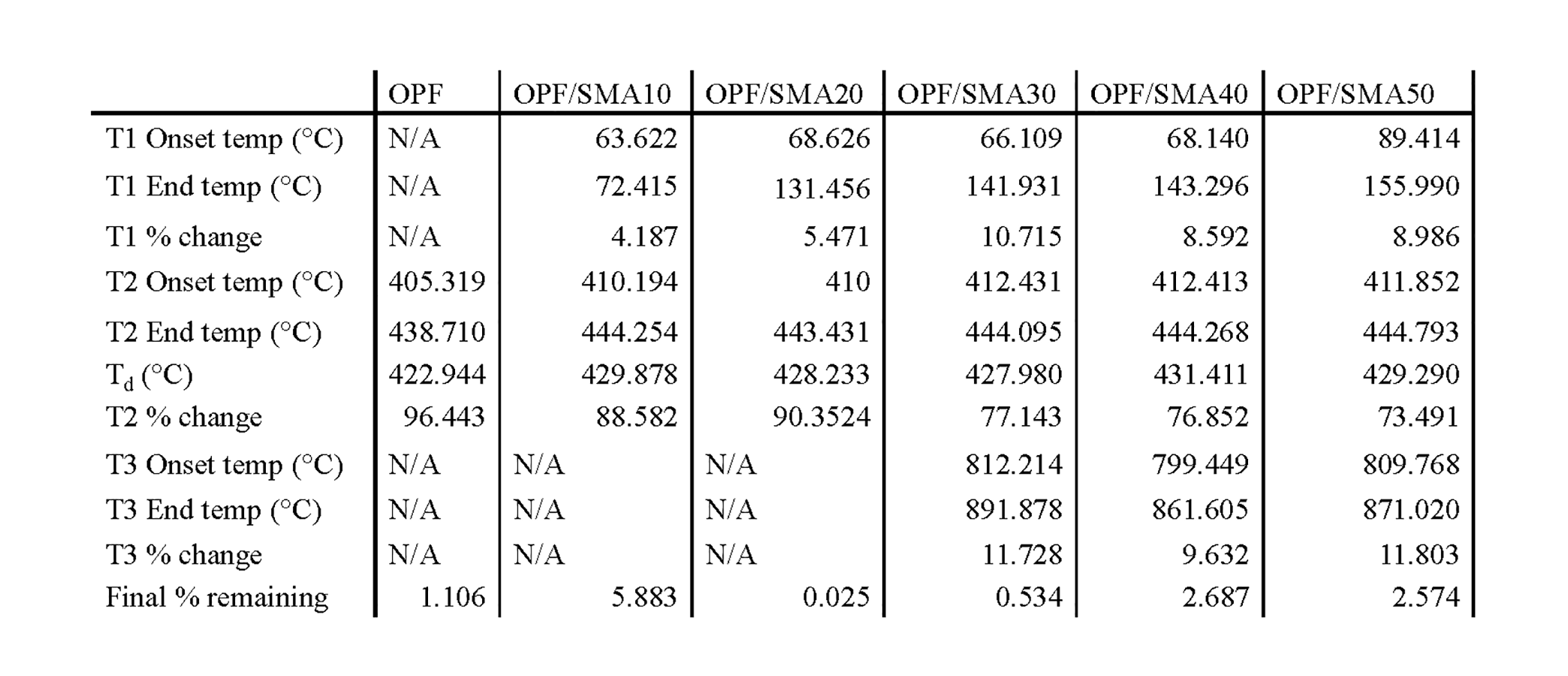

Supplement: S1 Table — Analysis of TGA scans and compilation of average degradation temperatures. Values are a mean calculated from replicate experiments (N = 2). Undetectable value denoted by (N/A). (TIF) [file pone.0146401.s004.tif]
